# Variable Speed Across Dimensions of Ability in the Joint Model for Responses and Response Times

**DOI:** 10.3389/fpsyg.2021.469196

**Published:** 2021-03-29

**Authors:** Peida Zhan, Hong Jiao, Kaiwen Man, Wen-Chung Wang, Keren He

**Affiliations:** ^1^Zhejiang Normal University, Jinhua, China; ^2^University of Maryland, College Park, MD, United States; ^3^University of Alabama, Tuscaloosa, AL, United States; ^4^The Education University of Hong Kong, Tai Po, Hong Kong

**Keywords:** response times, joint model, variable speed, multidimensional item response theory, hierarchical modeling framework

## Abstract

Working speed as a latent variable reflects a respondent’s efficiency to apply a specific skill, or a piece of knowledge to solve a problem. In this study, the common assumption of many response time models is relaxed in which respondents work with a constant speed across all test items. It is more likely that respondents work with different speed levels across items, in specific when these items measure different dimensions of ability in a multidimensional test. Multiple speed factors are used to model the speed process by allowing speed to vary across different domains of ability. A joint model for multidimensional abilities and multifactor speed is proposed. Real response time data are analyzed with an exploratory factor analysis as an example to uncover the complex structure of working speed. The feasibility of the proposed model is examined using simulation data. An empirical example with responses and response times is presented to illustrate the proposed model’s applicability and rationality.

## Introduction

With the popularity of computer-based tests, the collection of item response times (RTs) has become a routine activity in large- and small-scale educational assessments. For example, the Programme for International Student Assessment (PISA) started using computer-based tests and recorded RTs data since 2012. RTs provide information about the working speed of respondents but also could be utilized to improve measurement accuracy because RTs are considered to convey a more synoptic depiction of the respondents’ performance beyond what is obtainable based on correct responses alone (van der Linden et al., 2010; Bolsinova and Tijmstra, 2018).

Before making inferences by employing RTs, it is necessary to create an appropriate statistical model for RTs. Over the past few decades, various RT models have been presented based on cognitive/psychological theories and experimental research (for a review, see De Boeck and Jeon, 2019). Currently, the Bayesian hierarchical modeling framework (van der Linden, 2007) is one of the most flexible tools to explain the relationship between latent ability and working speed. This framework is gaining more recognition and is sufficiently generalized to integrate available measurement models for item response accuracy (RA) and RTs. Typically, in the hierarchical modeling of RTs and RA, the RT measurement model assumes that a respondent works at a constant speed throughout a test. Meanwhile, the RA measurement model assumes that a respondent puts his or her best effort forward to solve a set of items correctly by using the required knowledge. Thus, the association between latent ability and working speed is assumed to be changeless for each respondent working on a test. In other words, each respondent is assumed to work at a constant pace given his or her invariant ability at that time (Fox and Marianti, 2016).

Currently, most joint models for RA and RTs only use unidimensional measurement models to capture the relationship between latent ability and working speed within a unidimensional test scenario (e.g., Klein Entink et al., 2009a,b; Wang et al., 2013; Fox et al., 2014; Molenaar et al., 2015, 2016; Wang and Xu, 2015; Fox and Marianti, 2016). In reality, however, multiple latent abilities are involved to correctly answer an item, especially in multidimensional tests (e.g., Tatsuoka, 1983; Reckase, 2009). Compared to unidimensional tests, one significant characteristic of multidimensional tests is that different test items may measure distinguish latent ability dimensions.

In educational and psychological measurements, working speed as a latent variable reflects a respondent’s efficiency to apply a specific skill or a piece of knowledge to solve a problem. Therefore, latent speed should be discussed by considering the linkage to a particular dimension of latent ability. It is reasonable to assume that respondents could vary their working speeds across items that measure different dimensions of ability. In other words, the multidimensional structure for latent ability could be used to model the process of speed change, where the working speed is allowed to vary across dimensions of ability. For example, in a math test, the working speed on items that measure algebra problem-solving ability may differ from those measuring geometry problem-solving ability.

With the development of psychometrics, multidimensional measurement models for RA [e.g., multidimensional item response theory (MIRT) models and diagnostic classification models (DCMs)] have been well developed and widely used (see Reckase, 2009; Rupp et al., 2010). Recently, based on hierarchical modeling, a few studies have attempted to use MIRT models or DCMs for RA to capture the multidimensional structure of the latent trait when multidimensional tests are involved. But still, a unidimensional or single-factor RT (SRT) model is used to measure latent speed (Zhan et al., 2018; Man et al., 2019; Wang et al., 2019). Thus, in these studies, the relationships between multiple latent abilities and one single latent speed are assumed to be constant for each respondent working with a constant speed on different items. However, assuming identical working speeds across different dimensions of ability may be too restrictive to describe intricate data and thus may lead to ambiguous conclusions. It is desirable to release this limitation to allow each dimension of ability to be associated with a specific speed factor. As current joint models may be inappropriate for multidimensional tests, it is critical to develop a joint model that allows working speed to vary across dimensions of ability.

To model varying working speeds within different domains of ability, it is possible to use multiple-speed factors/dimensions to describe the speed process. Each speed factor corresponds to a specific dimension of latent ability. An individual speed process is assumed, describing the changes in speed across dimensions. Thus, respondents can work at different levels of speed on items within different dimensions of ability during multidimensional tests. Each individual speed process will be defined using a confirmatory multifactor structure, which in turn is defined by the dimensions of ability measured by items, according to the testing blueprint. Furthermore, it will be shown that the multifactor working speed model can be integrated with a MIRT model for latent ability. Under this new joint model, it is assumed that each respondent works at a unique speed corresponding to the dimension represented by an item.

We first extend the most popular single-factor lognormal RT (SLRT) model (van der Linden, 2006) to a multifactor working speed model that considers changing speed across dimensions. This is called the multifactor lognormal RT (MLRT) model. Second, a joint model of multidimensional latent ability and multifactor working speed will be proposed. Our paper starts with a brief review of the SLRT model, followed by presenting the proposed MLRT model. The proposed joint model is then presented. Next, a motivating example will be provided to demonstrate the multifactor structure of working speed and its compatibility with the multidimensional structure of latent ability. Moreover, two simulation studies will be conducted to evaluate the psychometric properties of the proposed joint model. An empirical example will also be analyzed to illustrate the application of the proposed joint model. Finally, we summarize our findings and discuss directions for future research.

## Multifactor Lognormal Response Time Model

Let *T*_*ni*_ be the observed RT of person *n* (*n* = 1,…, *N*) to item *i* (*i* = 1,…, *I*). In the SLRT model, the logarithmic function is used to transform the positively skewed distribution of RT to a more symmetric shape and is assumed to be dominated by item *i*’s time-intensity parameter ξ*_*i*_* and person *n*’s latent speed parameter τ*_*n*_* as follows:

(1) $$\log T_{n\,i}=\xi_{i}-\tau_{n}+\varepsilon_{n\,i},\varepsilon_{n\,i}∼N\,\left(0,\omega_{i}^{-2}\right),$$

or equivalently,

(2) $$\log T_{n\,i}∼N\,\left(\xi_{i}-\tau_{n},\omega_{i}^{-2}\right).$$

where **ξ**_*i*_ represents the time needed to complete item *i*, τ_*n*_ is the single-factor working speed of person *n*, and ε*_*ni*_* is the normally distributed residual error term, with mean zero and variance$\omega_{i}^{-2}$, where ω*_*i*_* is the time-precision parameter.

In recent years, the SLRT model has been extended in some studies. For instance, Klein Entink et al. (2009a) included a time-discrimination parameter as a slope parameter for latent speed. Klein Entink et al. (2009b) proposed the Box-Cox transformation for RT modeling. Wang et al. (2013) proposed a linear transformation model for RTs. Furthermore, Fox and Marianti (2016) proposed a variable working speed model, which allows the respondents to adjust their working speed along the sequence of items throughout the test. Although Fox and Marianti’s (2016) model relaxed the assumption of constant speed in the SLRT model, their variable speed was different from that focused on in this study. One is to change speed as the item response progresses, and the other is to change speed as the dimension of ability examined by the item changes.

As mentioned previously, the kernel hypothesis of this study is that respondents can work with different levels of speed on items requiring different dimensions of ability during multidimensional tests. In other words, working speed has a multifactor structure, which is defined by the multidimensional structure of ability. In the multidimensional test, assuming there are *K* sub-dimensions of latent ability. In the current study, only the between-item multidimensionality (Adams et al., 1997) is considered, where each item measures a single dimension but different items measure different dimensions, so the multidimensionality occurs between items. To model variable speed across dimensions, we first relaxed the assumption of the SLRT model that each respondent works at a constant speed on all items throughout the test and allowed the instantaneous speed to be different on different items, that is, τ*_*n*_*→${\tilde{\tau }}_{n\,i}$. Then, a confirmatory multifactor structure was given to model the instantaneous speed at item *i* of person *n*, as

(3) $${\tilde{\tau }}_{n\,i}=\sum_{k=1}^{K}\tau_{n\,k}\,q_{i\,k},$$

where ${\tilde{\tau }}_{n\,i}$ is the instantaneous speed at item *i* of person *n*, and τ*_*nk*_* is the working speed factor of person *n* corresponding to *k*th-dimension (*k* = 1, 2,…, *K*) of ability. The Q-matrix (Tatsuoka, 1983) is an *I*-by-*K* confirmatory matrix with element *q*_*ik*_ indicating whether *k*th-dimension of ability is required to answer item *i* correctly: *q*_*ik*_ = 1 if the dimension is required, and *q*_*ik*_ = 0 otherwise. For between-item multidimensionality, only one dimension is measured by an item, namely, only one element in **q***_*i*_* equals to 1. In such cases, the MLRT model can be expressed as

(4) $$\log T_{n\,i}=\xi_{i}-{\tilde{\tau }}_{n\,i}+\varepsilon_{n\,i}=\xi_{i}-\sum_{k=1}^{K}\tau_{n\,k}\,q_{i\,k}+\varepsilon_{n\,i},\varepsilon_{n\,i}∼N\,\left(0,\omega_{i}^{-2}\right)$$

or equivalently,

(5) $$\log T_{n\,i}∼N\,\left(\xi_{i}-{\tilde{\tau }}_{n\,i},\omega_{i}^{-2}\right).$$

If only one dimension of ability is assumed to be measured by all items, the MLRT model reduces to the SLRT model.

## Joint Model for Response Accuracy and Response Times

### Model Construction

Since both RA and RTs contain information about items and persons, it is advantageous to analyze them simultaneously. To this end, based on hierarchical modeling, we propose a new joint model called the multidimensional-multifactor joint (MMJ) model. For illustration purposes, in the MMJ model in this study, the MLRT model is used as the measurement model for RTs, and according to the 2012 PISA mathematics assessment framework (OECD, 2013), the multidimensional Rasch (MR) model (Adams et al., 1997) is employed as the measurement model for RA.

Besides observing RTs, let *Y*_*ni*_ be the observed RA for person *n* to item *i.* The MR model can be expressed as

(6) $$\text{logit}\left(P\left(Y_{n\,i}=1\right)\right)=\sum_{k=1}^{K}\theta_{n\,k}q_{i\,k}+d_{i},$$

where logit(*x*) = log(*x*/(1–*x*)), *P*(*Y*_*ni*_ = 1) is the probability of a correct response by person *n* to item *i*, θ*_*nk*_* is the latent ability of person *n* on dimension *k*, *d*_*i*_ is the intercept or easiness of item *i*, and *q*_*ik*_ is the element of Q-matrix.

The multivariate normal distribution was used to describe the relationships among the multidimensional ability and multifactor speed:

$$\left(\begin{array}{c} \theta_{n}\\ \tau_{n} \end{array}\right)\,\text{∼}\,N\,\left(\left(\begin{array}{c} \mu_{\theta }\\ \mu_{\tau } \end{array}\right),\,\sum_{\text{Person}}\right),$$

(7) $$\sum_{\text{Person}}=\left(\begin{array}{cccccc} \sigma_{\theta_{1}}^{2} & & & & & \\ \vdots & \ddots & & & & \\ \sigma_{\theta_{1}\,\theta_{K}} & \cdots & \sigma_{\theta_{K}}^{2} & & & \\ \sigma_{\theta_{1}\,\tau_{1}} & \cdots & \sigma_{\theta_{K}\,\tau_{1}} & \sigma_{\tau_{1}}^{2} & & \\ \vdots & \cdots & \vdots & \vdots & \ddots & \\ \sigma_{\theta_{1}\,\tau_{K}} & \cdots & \sigma_{\theta_{K}\,\tau_{K}} & \sigma_{\tau_{1}\,\tau_{K}} & \cdots & \sigma_{\tau_{K}}^{2} \end{array}\right),$$

where θ*_*n*_* = (θ*_*n*_*_1_,…, θ*_*nk*_*,…, θ*_*nK*_*)’ is the multidimensional latent ability vector; τ_*n*_ = (τ_*n1*_,…,τ_*nk*_,…,τ*_*nK*_*)’ is the multifactor working speed vector; μ_θ_ and μ_τ_ are the population mean vector of multidimensional ability and the population mean vector of multifactor working speed, respectively; and **Σ**_*person*_ is a variance-covariance matrix of person parameters, where $\sigma_{\theta_{k}}^{2}$is the variance of θ*_*k*_*, $\sigma_{\tau_{k}}^{2}$is the variance of τ*_*k*_*, σ_*θ_k θ_k’*_is the covariance of θ*_*k*_* and θ*_*k*__’_*, σ_*τ_k τ_k’*_is the covariance of τ*_*k*_* and τ*_*k*__’_*, and σ_*τ_k τ_k’*_is the covariance of θ*_*k*_* and τ*_*k*_*.

Furthermore, for the item parameters, a bivariate normal distribution was used to describe the relationship between item easiness and item time-intensity,

(8) $$\left(\begin{array}{c} d_{i}\\ \xi_{i} \end{array}\right)∼N\,\left(\left(\begin{array}{c} \mu_{d}\\ \mu_{\xi } \end{array}\right),\sum_{\text{item}}\right),\sum_{\text{item}}=\left(\begin{array}{cc} \sigma_{d}^{2} & \\ \sigma_{d\,\xi } & \sigma_{\xi }^{2} \end{array}\right),$$

where μ*_*d*_* and μ_ξ_ are the mean of item easiness and the mean of item time-intensity, respectively; and **Σ**_*item*_ is a variance-covariance matrix of item parameters, where $\sigma_{d}^{2}$ and $\sigma_{\xi }^{2}$ are the variance of item easiness and the variance of item time-intensity, respectively; σ_*d*ξ**_is the covariance of item easiness and item time-intensity.. The residual error variance, $\omega_{i}^{-2}$, is assumed to be independently distributed.

For the MMJ model, the latent scales of multidimensional ability and mutlifactor speed need to be identified. This can be accomplished by restricting the population mean of the ability and speed as μ_θ_ = μ_τ_ = **0**.

### Parameter Estimation

Parameters in the MMJ model can be estimated via the full Bayesian approach with the Markov Chain Monte Carlo (MCMC) method. In Bayesian estimation, prior distributions of model parameters and observed data likelihood produce a joint posterior distribution for the model parameters. In this study, the Just Another Gibbs Sampler (JAGS) software (Plummer, 2015) was used to estimate parameters. JAGS uses a default option of the Gibbs sampler (Gelfand and Smith, 1990), whose code for the proposed joint model is provided in the online Supplementary Appendix.

Under the assumption of local independence, *Y*_*ni*_ and log*T*_*ni*_ are independently distributed as

$$Y_{n\,i}∼\text{Bernoulli}\left(P\left(Y_{n\,i}=1\right)\right)and\log T_{n\,i}∼N\left(\xi_{i}-{\tilde{\tau }}_{n\,i},\omega_{i}^{-2}\right).$$

Weakly but not non-informative priors are preferentially used in this study to increase the generalizability of our codes by imposing vague prior beliefs on estimating parameters. The setting of priors refers to that used by Zhan et al. (2018) and Man et al. (2019).

The priors of the person parameters are set as

$$\left(\begin{array}{c} \theta_{n}\\ \tau_{n} \end{array}\right)∼N\,\left(\left(\begin{array}{c} 0\\ 0 \end{array}\right),\sum_{\text{person}}\right),$$

with a hyper prior

$$\sum_{\text{person}}∼\mathrm{InvWishart}\,\left({\text{R}}_{\text{person}},K^{*}\right),$$

where **R**_*person*_ is a K*-dimensional identity matrix, and K* indicates the degree of freedom, which in this case is equal to the dimension of the **R**_*person*_.

In addition, the priors of item parameters are set as

$$\left(\begin{array}{c} d_{i}\\ \xi_{i} \end{array}\right)∼N\,\left(\left(\begin{array}{c} \mu_{d}\\ \mu_{\xi } \end{array}\right),\sum_{\text{item}}\right),\omega_{i}^{-2}∼\text{InvGamma}\,\left(1,1\right)$$

.

Furthermore, the hyper priors are specified as

$$\mu_{d}∼\text{Normal}\,\left(0,2\right),\mu_{\xi }∼\text{Normal}\,\left(4.3,2\right),$$

$$\sum_{i\,t\,e\,m}∼\mathrm{InvWishart}\,\left(R_{\text{item}},2\right),$$

where **R**_*item*_ is a two-dimensional identity matrix. Finally, the posterior mean is treated as the estimated value for model parameters.

## A Motivating Example

To explore the multifactor structure of working speed, and to explore whether this structure matches the multidimensional structure of latent ability, a motivating example with the exploratory factor analysis (EFA) of RTs was presented first.

### Data Description

The PISA 2012 computer-based mathematics RT data were analyzed. This data set was originally used by Zhan et al. (2018). In this study, there are *N* = 1,581 respondents and *I* = 9 items. The logarithm of RTs was computed before the analysis, and all zero RTs were treated as missing data. A Q-matrix (see Table 1) was specified based on the PISA 2012 mathematics assessment framework (OECD, 2013). Three dimensions that belong to the mathematical content knowledge were chosen, namely, change and relationships (θ_1_), space and shape (θ_2_), and uncertainty and data (θ_3_). However, it should be noted that this Q-matrix was originally used to link items and latent abilities or to present the multidimensional structure of latent ability. In other words, this Q-matrix does not specify the latent structure of working speed unless the structure explored by the EFA of RTs matches it.

**TABLE 1:** Q-Matrix for PISA 2012 released computer-based mathematics items.

**Items**	**θ_1_**	**θ_2_**	**θ_3_**
CM015Q02D	1		
CM015Q03D	1		
CM020Q01		1	
CM020Q02		1	
CM020Q03		1	
CM020Q04		1	
CM038Q03T			1
CM038Q05			1
CM038Q06			1

*Blank means zero.*

### Exploratory Analysis and Results

The Mplus (version 8.1) (Muthén and Muthén, 2019) was used here. The EFA within a confirmatory factor analysis framework method was used by default in Mplus. In this study, the number of factors to retain was set as 1 to 5, which means 1- to 5-factor CFA models were all employed to fit RT data. Then, Akaike Information Criterion (AIC; Akaike, 1974) and Bayesian Information Criterion (BIC; Schwarz, 1978) were used as model-data fit indexes to help judge the number of factors/dimensions. Theoretically, correlations should exist among multiple dimensions; thus, oblique rotation was used. Other settings followed the default (e.g., the maximum likelihood was used as an extraction method).

Table 2 presents the model-data fit indexes of the EFA. According to previous studies, TLI > 0.95, CFI > 0.95, SRMR ≤ 0.08, and RMSEA < 0.05 mean good model-data fit (Hu and Bentler, 1999; Steiger, 1990). The AIC preferred the 4-factor model, and the BIC preferred the 3-factor model after taking into account the penalty weighting of sample size. On the whole, the 3-factor model seems to fit the data better than the other models.

**TABLE 2:** Exploratory factor analysis model-data fit indexes for RT data.

**Model**	**χ^2^**	***df***	**TLI**	**CFI**	**AIC**	**BIC**	**SRMR**	**RMSEA (90% CI)**
1-factor	462.79**	27	0.896	0.922	24592.15	24737.03	0.045	0.101 (0.093, 0.109)
2-factor	225.49**	19	0.930	0.963	24370.85	24558.65	0.032	0.083 (0.073, 0.093)
3-factor	32.66**	12	0.989	0.996	24192.02	24417.38	0.010	0.033 (0.020, 0.047)
4-factor	5.56	6	1.000	1.000	24176.92	24434.48	0.004	0.000 (0.000, 0.031)
5-factor	0.09	1	1.006	1.000	24181.44	24465.83	0.000	0.000 (0.000, 0.045)

**p < 0.01; χ^2^ = chi-square; *df* = degrees of freedom; TLI = Tucker-Lewis index; CFI = comparative fit index; AIC = Akaike information criterion; BIC = Bayesian information criterion; SRMR = standardized root mean square residual; RMSEA = root mean square error of approximation; CI = confidence interval.

Table 3 presents the rotated factor loading matrix for the 3-factor model. Compared to the theoretically constructed Q-matrix for latent ability, there is only a difference in CM038Q03T. The rotated factor loading of CM038Q03T on Factor 3 is 0.300 (*p* < 0.05), which also supports the theoretical structure to a certain extent. The results indicate that the latent structure of working speed might be a 3-factor structure, which is also consistent with the theoretical multidimensional structure of latent ability (i.e., the Q-matrix in Table 1).

**TABLE 3:** Rotated factor loading matrix for the 3-factor model for response times data.

**Item**	**Factor 1**	**Factor 2**	**Factor 3**
CM015Q02D	0.695*		
CM015Q03D	0.609*		
CM020Q01		0.565*	
CM020Q02		0.801*	
CM020Q03		0.642*	
CM020Q04		0.943*	
CM038Q03T		0.502*	
CM038Q05			0.985*
CM038Q06			0.621*

*p < 0.05; absolute value of factor loading below 0.4 was omitted.

Overall, the results of the EFA support the kernel hypothesis of this study. However, due to the limitations of the EFA, the estimation of parameters such as individual working speed cannot be realized. Therefore, further exploration and utilization of the proposed MMJ model are necessary.

## Simulation Studies

Two simulation studies were conducted to evaluate the performance of the MMJ model under various conditions. The primary purpose of simulation study 1 was to examine whether the model parameters could be recovered accurately using the proposed Bayesian estimation algorithm, in which data were simulated from the MMJ model and analyzed with itself.

Man et al. (2019) has shown that, in multidimensional tests, the joint model that involves multidimensional ability and single-factor speed (denoted as MSJ model in this study) performs better than the joint model that involves unidimensional ability and single-factor speed (e.g., van der Linden, 2007). In this study, we focus on the comparison between the MMJ model and the MSJ model. Specifically, simulation study 2 was conducted to evaluate: (a) the consequences of ignoring the multifactor structure of working speed, in which the data were simulated from the MMJ model but analyzed with the MSJ model; and (b) the consequences of misspecifying a multifactor structure of working speed, in which the data were simulated from the MSJ model but analyzed with the MMJ model. Note that the results of simulation study 2 were omitted for brevity but can be found in the online Supplementary Appendix (see Supplementary section S1).

### Design and Data Generation

**FIGURE 1:**
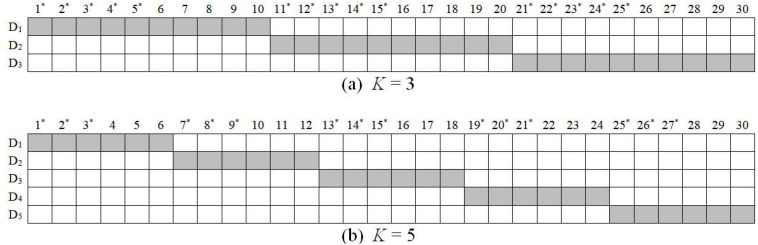
*K-by-I Q’ matrix in the simulation study 1. D* = dimension of latent ability; items with * are used for *I* = 15 conditions.

In simulation study 1, four factors were manipulated including (a) sample size: *N* = 500 and 1,000, (b) test length: *I* = 15 and 30, (c) the correlation coefficient between latent ability and its corresponding working speed factor: ρ_θ__τ_ = –0.7 and –0.4, and (d) the number of dimensions of ability: *K* = 3 and 5. Q-matrices are presented in Figure 1. In addition, the true values of other parameters were generated according to the results of a data analysis using real data (Zhan et al., 2018). For item parameters, item easiness, *d*_*i*_, and item time intensity, ξ*_*i*_*, were generated from a bivariate normal distribution with mean vector (0, 4) and covariance matrix of [1, –0.2; –0.2, 0.25]. In such a setting, ρ*_*d*_*_ξ_ = –0.4. The reciprocal of the standard deviation of the error term, ω, is set to 2 for all items. Person parameters were generated from${\left(\theta_{n},\,\tau_{n}\right)}^{\prime }∼N\,\left({\left(0,\,0\right)}^{\prime },\sum_{P\,e\,r\,s\,o\,n}\right)$, where

$$\sum_{P\,e\,r\,s\,o\,n}=\left(\begin{array}{cccccc} \sigma_{\theta_{1}}^{2} & & & & & \\ \vdots & \ddots & & & & \\ \sigma_{\theta_{1}\,\theta_{K}} & \cdots & \sigma_{\theta_{K}}^{2} & & & \\ \sigma_{\theta_{1}\,\tau_{1}} & \cdots & \sigma_{\theta_{K}\,\tau_{1}} & \sigma_{\tau_{1}}^{2} & & \\ \vdots & \cdots & \vdots & \vdots & \ddots & \\ \sigma_{\theta_{1}\,\tau_{K}} & \cdots & \sigma_{\theta_{K}\,\tau_{K}} & \sigma_{\tau_{1}\,\tau_{K}} & \cdots & \sigma_{\tau_{K}}^{2} \end{array}\right)$$

$$=\left(\begin{array}{cccccc} 1 & & & & & \\ \vdots & \ddots & & & & \\ 0.8 & \cdots & 1 & & & \\ 0.5\,\rho_{\theta \,\tau } & \cdots & 0.5\,\rho_{\theta \,\tau } & 0.25 & & \\ \vdots & \ddots & \vdots & \vdots & \ddots & \\ 0.5\,\rho_{\theta \,\tau } & \cdots & 0.5\,\rho_{\theta \,\tau } & 0.15 & \cdots & 0.25 \end{array}\right).$$

In such a case, the covariance of two latent abilities is σ_θ__θ__’_ = 0.8 (i.e., correlation coefficient ρ_θ__θ__’_ = 0.8) and the covariance of two latent speeds is σ_τ τ ’_ = 0.15 (i.e., correlation coefficient ρ_τ τ ’_ = 0.6). Thirty data sets were generated.

### Analysis

In simulation study 1, the MMJ model was fitted to each of the 30 replications. In each replication, two Markov chains with random starting points were used, and each chain ran 10,000 iterations with the first 5,000 iterations in each chain as burn-in. Finally, the remaining 10,000 iterations were used for the model parameter inferences. The potential scale reduction factor (PSRF; Brooks and Gelman, 1998) was computed to assess the convergence of each parameter. A PSRF with values smaller than 1.2 indicates convergence. Our studies indicated that the PSRF was smaller than 1.1 for all parameters, suggesting good convergence.

To evaluate parameter recovery, the bias and the root mean square error (RMSE) was computed as: $\left(\hat{\upsilon }\right)=\sum_{r=1}^{R}\frac{\hat{\upsilon }-\upsilon }{R}$ and $\text{RMSE}\,\left(\hat{\upsilon }\right)=\sqrt{\sum_{r=1}^{R}\frac{{\left(\hat{\upsilon }-\upsilon \right)}^{2}}{R}}$, where ${\hat{\upsilon }}_{r}$ is the estimated value of the model parameter in *r*th replication and υ is the true value of the corresponding model parameter, respectively; *R* is the total number of replications. The correlation between estimated and true values (Cor) was also computed.

### Results

Table 4 presents the recovery of item parameters. All item parameters were well recovered. The recovery of time-intensity was the best, followed by time-discrimination, and then item easiness. An increasing sample size yielded a better recovery of item parameters. It seems that test length, the correlation coefficient between latent ability and latent speed, and the number of dimensions have a limited impact on the recovery of item parameters.

**TABLE 4:** Recovery of item parameters in simulation study 1.

				**Mean Bias**			**Mean RMSE**			**Cor**		
***I***	***N***	**ρ_θ__τ_**	***K***	***d***	**ξ**	**ω**	***d***	**ξ**	**ω**	***d***	**ξ**	**ω**
15	500	−0.4	3	0.000	0.001	−0.013	0.106	0.021	0.075	0.995	0.999	NA
			5	0.011	–0.001	−0.024	0.110	0.023	0.077	0.995	0.999	NA
		−0.7	3	−0.006	0.000	−0.016	0.098	0.024	0.073	0.996	0.999	NA
			5	0.009	0.001	−0.017	0.114	0.022	0.085	0.994	0.999	NA
	1000	−0.4	3	−0.001	−0.001	−0.009	0.076	0.016	0.051	0.997	1.000	NA
			5	0.001	0.001	−0.012	0.074	0.015	0.056	0.998	1.000	NA
		−0.7	3	–0.002	0.000	−0.011	0.077	0.015	0.052	0.997	1.000	NA
			5	0.002	0.000	−0.014	0.077	0.016	0.053	0.997	1.000	NA
30	500	−0.4	3	–0.006	0.000	−0.015	0.110	0.022	0.070	0.994	0.999	NA
			5	0.003	0.000	−0.018	0.106	0.022	0.073	0.995	0.999	NA
		−0.7	3	−0.001	–0.001	−0.017	0.103	0.022	0.067	0.995	0.999	NA
			5	−0.003	0.000	−0.019	0.106	0.023	0.074	0.995	0.999	NA
	1000	−0.4	3	0.001	–0.001	−0.007	0.075	0.016	0.047	0.997	1.000	NA
			5	−0.003	0.000	−0.007	0.076	0.015	0.051	0.997	1.000	NA
		−0.7	3	0.000	0.000	−0.008	0.077	0.016	0.050	0.997	0.999	NA
			5	−0.002	0.000	−0.010	0.076	0.016	0.051	0.997	1.000	NA

**I* = test length; *N* = sample size; ρ_θ__τ_ = correlation coefficient between ability and speed; *K* = number of dimensions of ability; *d* = item easiness; ξ = item time-intensity; *ω* = item time-discrimination; Mean Bias = mean bias across all items; Mean RMSE = mean root mean square error across all items; Cor = correlation between estimated and true values. Cor of ω is NA because of the variance of true *ω* is zero.*

Tables 5, 6 present the recovery of ability and speed, respectively. First, the recovery of multiple speed factors was better than that of abilities. Increasing test length yielded a better recovery of person parameters; by contrast, increasing the number of dimensions yielded a worse recovery of person parameters. In addition, the higher the correlation coefficient between ability and speed, the better the recovery of latent abilities becomes; however, the correlation coefficient had little effect on the recovery of latent speeds.

**TABLE 5:** Recovery of multidimensional ability in simulation study 1.

				**Mean Bias**					**Mean RMSE**					**Cor**				
***I***	***N***	**ρ_θ__τ_**	***K***	**θ_1_**	**θ_2_**	**θ_3_**	**θ_4_**	**θ_5_**	**θ_1_**	**θ_2_**	**θ_3_**	**θ_4_**	**θ_5_**	**θ_1_**	**θ_2_**	**θ_3_**	**θ_4_**	**θ_5_**
15	500	−0.4	3	0.000	0.000	0.000			0.599	0.599	0.598			0.798	0.800	0.800		
			5	0.000	0.000	0.000	0.000	0.000	0.623	0.627	0.624	0.625	0.623	0.780	0.779	0.782	0.781	0.781
		−0.7	3	0.000	0.000	0.000			0.520	0.518	0.519			0.854	0.854	0.854		
			5	0.001	0.000	0.000	0.000	0.000	0.522	0.529	0.526	0.523	0.524	0.853	0.849	0.851	0.853	0.850
	1000	−0.4	3	0.000	0.000	0.000			0.592	0.592	0.594			0.803	0.803	0.802		
			5	0.000	0.000	0.000	0.000	0.000	0.615	0.617	0.619	0.618	0.617	0.786	0.785	0.783	0.783	0.785
		−0.7	3	0.000	0.000	0.000			0.515	0.514	0.514			0.856	0.856	0.856		
			5	0.000	0.000	0.000	0.000	0.000	0.519	0.522	0.522	0.524	0.519	0.854	0.852	0.851	0.850	0.854
30	500	−0.4	3	0.000	0.000	0.000			0.497	0.495	0.497			0.866	0.867	0.866		
			5	0.000	0.000	0.000	0.000	0.000	0.540	0.536	0.536	0.534	0.526	0.840	0.842	0.843	0.844	0.849
		−0.7	3	0.000	0.000	0.000			0.448	0.450	0.449			0.893	0.892	0.892		
			5	0.000	0.000	0.000	0.000	0.000	0.474	0.474	0.470	0.473	0.470	0.879	0.880	0.881	0.880	0.881
	1000	−0.4	3	0.000	0.000	0.000			0.491	0.489	0.490			0.869	0.870	0.869		
			5	0.000	0.000	0.000	0.000	0.000	0.528	0.528	0.526	0.529	0.529	0.846	0.847	0.848	0.847	0.846
		−0.7	3	0.000	0.000	0.000			0.447	0.450	0.448			0.892	0.892	0.892		
			5	0.000	0.000	0.000	0.000	0.000	0.469	0.468	0.473	0.470	0.470	0.882	0.883	0.880	0.882	0.881

*I = test length; N = sample size; ρ_θ__τ_ = correlation coefficient between ability and speed; K = number of dimensions of ability; θ = latent ability; Mean Bias = mean bias across all persons; Mean RMSE = mean root mean square error across all persons; Cor = correlation between estimated and true values.*

**TABLE 6:** Recovery of multifactor speed in simulation study 1.

				**Mean Bias**					**Mean RMSE**					**Cor**				
**I**	**N**	**ρθ τ**	**K**	τ**_1_**	τ**_2_**	τ**_3_**	τ**_4_**	τ**_5_**	τ**_1_**	τ**_2_**	τ**_3_**	τ**_4_**	τ**_5_**	τ**_1_**	τ**_2_**	τ**_3_**	τ**_4_**	τ**_5_**
15	500	−0.4	3	0.000	0.000	0.000			0.191	0.194	0.194			0.922	0.920	0.920		
			5	0.000	0.000	0.000	0.000	0.000	0.225	0.225	0.227	0.227	0.226	0.891	0.888	0.890	0.889	0.890
		−0.7	3	0.000	0.000	0.000			0.191	0.190	0.188			0.922	0.924	0.925		
			5	0.000	0.000	0.000	0.000	0.000	0.221	0.221	0.221	0.224	0.224	0.895	0.895	0.895	0.892	0.893
	1000	−0.4	3	0.000	0.000	0.000			0.193	0.191	0.193			0.921	0.923	0.921		
			5	0.000	0.000	0.000	0.000	0.000	0.226	0.226	0.224	0.227	0.224	0.890	0.891	0.892	0.889	0.892
		−0.7	3	0.000	0.000	0.000			0.189	0.189	0.189			0.925	0.925	0.925		
			5	0.000	0.000	0.000	0.000	0.000	0.221	0.221	0.221	0.223	0.223	0.895	0.894	0.895	0.894	0.894
30	500	−0.4	3	0.000	0.000	0.000			0.144	0.145	0.146			0.957	0.956	0.956		
			5	0.000	0.000	0.000	0.000	0.001	0.177	0.177	0.177	0.176	0.177	0.934	0.935	0.934	0.935	0.934
		−0.7	3	0.000	0.000	0.000			0.143	0.144	0.144			0.957	0.957	0.957		
			5	0.000	0.000	0.000	0.000	0.000	0.175	0.175	0.176	0.173	0.175	0.935	0.936	0.935	0.937	0.936
	1000	−0.4	3	0.000	0.000	0.000			0.144	0.144	0.146			0.957	0.957	0.956		
			5	0.000	0.000	0.000	0.000	0.000	0.175	0.175	0.176	0.177	0.176	0.936	0.935	0.935	0.934	0.935
		−0.7	3	0.000	0.000	0.000			0.142	0.143	0.144			0.958	0.958	0.957		
			5	0.000	0.000	0.000	0.000	0.000	0.174	0.174	0.173	0.174	0.174	0.936	0.936	0.937	0.937	0.936

*I = test length; N = sample size; ρ_θ__τ_ = correlation coefficient between ability and speed; K = number of dimensions of ability; τ = speed factor; Mean Bias = mean bias across all persons; Mean RMSE = mean root mean square error across all persons; Cor = correlation between estimated and true values.*

Table 7 presents the recovery of the item mean vector and item variance-covariance. Increasing test length and sample size yielded a better recovery. However, the correlation coefficient between ability and speed and the number of dimensions had a limited effect on the recovery. Additionally, the recovery of covariances (omitted, due to space limitations) was better than that of variances of item parameters.

**TABLE 7:** Recovery of item mean vector and item variance-covariance in simulation study 1.

				**Bias**					**RMSE**				
**I**	**N**	**ρθ τ**	**K**	σ**_d_^2^**	σ**_d__ξ_**	σ**_ξ_^2^**	μ**_d_**	μ**_ξ_**	σ**_d_^2^**	σ**_d__ξ_**	σ**_ξ_^2^**	μ**_d_**	μ**_ξ_**
15	500	−0.4	3	0.155	0.021	0.095	0.020	0.006	0.169	0.027	0.096	0.027	0.007
			5	0.135	0.013	0.097	0.023	0.005	0.162	0.016	0.097	0.029	0.007
		−0.7	3	0.151	0.016	0.095	0.024	0.006	0.164	0.020	0.095	0.029	0.007
			5	0.142	0.021	0.094	0.026	0.005	0.163	0.026	0.094	0.032	0.007
	1000	−0.4	3	0.158	0.015	0.096	0.013	0.005	0.164	0.018	0.096	0.016	0.006
			5	0.124	0.015	0.095	0.015	0.004	0.136	0.020	0.095	0.018	0.006
		−0.7	3	0.150	0.018	0.096	0.016	0.005	0.159	0.022	0.096	0.019	0.006
			5	0.132	0.016	0.096	0.018	0.004	0.144	0.019	0.096	0.021	0.004
30	500	−0.4	3	0.070	0.012	0.046	0.024	0.004	0.083	0.016	0.046	0.027	0.005
			5	0.060	0.012	0.044	0.018	0.003	0.068	0.014	0.045	0.023	0.004
		−0.7	3	0.078	0.010	0.046	0.013	0.004	0.090	0.012	0.046	0.016	0.005
			5	0.056	0.012	0.043	0.019	0.003	0.066	0.014	0.043	0.023	0.004
	1000	−0.4	3	0.062	0.006	0.044	0.013	0.003	0.069	0.008	0.044	0.016	0.004
			5	0.053	0.008	0.045	0.013	0.002	0.060	0.010	0.045	0.017	0.003
		−0.7	3	0.068	0.010	0.046	0.009	0.003	0.079	0.012	0.046	0.012	0.004
			5	0.044	0.007	0.046	0.012	0.002	0.052	0.009	0.046	0.015	0.003

*I = test length; N = sample size; ρ_θ__τ_ = correlation coefficient between ability and speed; K = number of dimensions of ability; σ_d_^2^ = variance of item easiness; σ_ξ_^2^ = variance of item time-intensity; σ_d__ξ_ = covariance of item easiness and item time-intensity; RMSE = mean root mean square error.*

Tables 8, 9 present the recovery of variances of person parameters. Similar to the pattern of the recovery of ability and speed, the recovery of variances of multiple speed factors was better than that of abilities. Increasing test length, sample size, and the correlation coefficient between ability and speed yielded a better parameter recovery. By contrast, more dimensions led to a worse recovery of variances of person parameters. Additionally, the recovery of covariances (omitted, due to space limitations) was better than that of variances of person parameters.

**TABLE 8:** Recovery of the variance of ability in simulation study 1.

				**Bias**					**RMSE**				
**I**	**N**	ρ**θ τ**	**K**	σ**_θ__1_^2^**	σ**_θ__2_^2^**	σ**_θ__3_^2^**	σ**_θ__4_^2^**	σ**_θ__5_^2^**	σ**_θ__1_^2^**	σ**_θ__2_^2^**	σ**_θ__3_^2^**	σ**_θ__4_^2^**	σ**_θ__5_^2^**
15	500	−0.4	3	0.002	−0.047	−0.026			0.152	0.154	0.142		
			5	−0.047	−0.088	−0.055	−0.056	−0.062	0.139	0.193	0.195	0.184	0.168
		−0.7	3	−0.007	0.000	0.010			0.140	0.121	0.142		
			5	−0.036	−0.066	−0.004	−0.016	−0.061	0.157	0.164	0.148	0.166	0.135
	1000	−0.4	3	−0.058	−0.033	−0.042			0.101	0.100	0.104		
			5	−0.072	−0.077	−0.023	−0.095	−0.092	0.123	0.147	0.116	0.139	0.140
		−0.7	3	−0.034	−0.018	−0.015			0.106	0.105	0.099		
			5	−0.071	−0.088	−0.067	−0.056	−0.045	0.148	0.139	0.117	0.131	0.118
30	500	−0.4	3	0.007	–0.035	0.010			0.090	0.099	0.078		
			5	−0.068	−0.085	−0.086	−0.054	−0.055	0.127	0.123	0.136	0.111	0.112
		−0.7	3	−0.014	−0.019	−0.017			0.082	0.097	0.080		
			5	−0.030	–0.075	−0.034	–0.070	−0.056	0.100	0.131	0.099	0.110	0.117
	1000	−0.4	3	−0.009	0.003	–0.040			0.060	0.057	0.063		
			5	−0.070	−0.033	−0.070	–0.084	–0.042	0.099	0.097	0.101	0.107	0.084
		−0.7	3	0.011	−0.032	−0.006			0.045	0.087	0.056		
			5	−0.050	−0.060	−0.069	−0.069	−0.072	0.100	0.091	0.110	0.113	0.101

*I = test length; N = sample size; ρ_θ__τ_ = correlation coefficient between ability and speed; K = number of dimensions of ability; σ_θ_^2^ = variance of ability; RMSE = mean root mean square error.*

**TABLE 9:** Recovery of the variance of speed factor in simulation study 1.

				**Bias**					**RMSE**				
**I**	**N**	**ρθ_τ_**	**K**	**σ_τ_1^2^**	**σ_τ_2^2^**	**σ_τ_3^2^**	**σ_τ_4^2^**	**σ_τ_5^2^**	**σ_τ_1^2^**	**σ_τ_2^2^**	**σ_τ_3^2^**	**σ_τ_4^2^**	**σ_τ_5^2^**
15	500	−0.4	3	0.002	0.001	0.004			0.010	0.010	0.010		
			5	0.004	–0.003	0.003	0.001	−0.001	0.012	0.016	0.017	0.013	0.013
		−0.7	3	0.001	0.002	0.002			0.011	0.009	0.010		
			5	0.007	0.002	0.002	0.003	0.001	0.015	0.014	0.013	0.012	0.015
	1000	−0.4	3	0.000	0.003	0.000			0.006	0.008	0.008		
			5	–0.003	0.005	0.000	−0.001	−0.001	0.009	0.013	0.011	0.011	0.008
		−0.7	3	0.002	0.001	0.001			0.009	0.007	0.008		
			5	0.001	–0.002	0.002	−0.002	0.003	0.010	0.010	0.009	0.011	0.009
30	500	−0.4	3	0.004	0.002	0.003			0.008	0.008	0.008		
			5	0.002	0.003	0.001	0.002	−0.001	0.009	0.008	0.009	0.009	0.009
		−0.7	3	0.003	0.002	0.005			0.007	0.008	0.011		
			5	0.003	0.001	0.004	0.002	0.001	0.010	0.009	0.010	0.010	0.010
	1000	–0.4	3	0.003	0.002	0.000			0.006	0.005	0.005		
			5	0.000	0.002	0.003	0.001	0.000	0.006	0.008	0.009	0.008	0.006
		−0.7	3	0.001	0.002	0.002			0.005	0.007	0.006		
			5	0.000	0.000	0.002	0.001	−0.001	0.008	0.006	0.006	0.007	0.006

*I = test length; N = sample size; ρ_θ__τ_ = correlation coefficient between ability and speed; K = number of dimensions of ability; σ_τ_^2^ = variance of speed factor; RMSE = mean root mean square error.*

In general, the recovery of time-related parameters (e.g., item intensity, the covariance of item easiness and time-intensity, speed factors, and covariance of ability and speed) was better than that of time-unrelated parameters (e.g., item easiness and latent abilities). Overall, simulation study 1 indicated that model parameters of the MMJ could be recovered very well via the proposed full Bayesian MCMC estimation algorithm.

## An Empirical Example

### Data Description and Analysis

In this section, the PISA 2012 computer-based mathematics RA and RT data were analyzed by using the MMJ model and the MSJ model to explore whether the former fits the data better than the latter when the test structure is multidimensional. Details about this data set were mentioned previously in the motivating example. The Q-matrix in Table 1 was used. For each model, in each replication, the numbers of chains, burn-in iterations, and post-burn-in iterations were the same as those set in the simulation study. Convergence was well achieved according to the PSRF < 1.1.

Posterior predictive model checking (PPMC; Gelman et al., 2014) was used to evaluate model-data fit. A posterior predictive probability (ppp) value near 0.5 indicates that there are no systematic differences between the realized and predictive values, and thus an adequate fit of the model. In PPMC, the sum of the squared Pearson residuals for person n and item i (Yan et al., 2003) was used as a discrepancy measure to evaluate the overall fit of the RA model, which is written as

$$D\,\left(Y_{n\,i};\theta_{n\,k},d_{i},q_{i\,k}\right)=\sum_{n=1}^{N}\sum_{i=1}^{I}{\left(\frac{Y_{n\,i}-P\left(Y_{n\,i}=1\right)}{\sqrt{P\left(Y_{n\,i}=1\right)\left(1-P\left(Y_{n\,i}=1\right)\right)}}\right)}^{2},$$

where *P*(*Y*_*ni*_ = 1) has the same definition as that in Equation (6). The sum of the standardized error function of log*T*_*ni*_ for person *n* and item *i* was employed as a discrepancy measure of the RT model:

$$D\,\left(\log T_{n\,i};\xi_{i},{\tilde{\tau }}_{n\,i},\omega_{i}\right)=\sum_{n=1}^{N}\sum_{i=1}^{I}{\left(\omega_{i}\,\left(\log T_{n\,i}-\left(\xi_{i}-{\tilde{\tau }}_{n\,i}\right)\right)\right)}^{2}.$$

Additionally, two information criteria that suitable for Bayesian estimation, the deviance information criterion (DIC) and widely available information criterion (WAIC) (Gelman et al., 2014, Chapter 7), were computed for model selection. A smaller value of these two criteria indicates a better model-data fit.

### Results

The DIC and WAIC both identified that the MMJ model fit the data better than the MSJ model, as shown in Table 10. In the MMJ model, the *ppp* values of the RA model and the RT model were 0.736 and 0.578, respectively, which indicates an adequate model-data fit. The results indicate that it is more appropriate to simultaneously consider the multidimensionality of latent ability and the multifactor structure of working speed for the multidimensional test.

**TABLE 10:** Model fit for the PISA 2012 computer-based mathematics data.

**Analysis Model**	**DIC**	**WAIC**	***ppp*_RA**	***ppp*_RT**
MMJ	**34853**	**34433**	0.736	0.578
MSJ	35910	35669	0.608	0.569

*MMJ, multidimensional-multifactor joint model; MSJ, multidimensional-single-factor joint model; DIC, deviance information criterion; WAIC, widely available information criterion; *ppp*, posterior predictive probability value; RA, item response accuracy; RT, item response times. Relatively smaller values are indicated in bold.*

Note that the parameter estimates of the MMJ model in the empirical example were omitted for brevity but can be found in the online Supplementary Appendix (see Supplementary section S2), mainly because this part of the content is not the main concern of the empirical study.

## Discussion

The kernel hypothesis of this study is that respondents can work with different levels of speed on items that require different dimensions of ability for a multidimensional test. To model the varying speed across dimensions of ability, this study relaxed the assumption of many RT models in which it is assumed that respondents work with a constant rate throughout the test. As a result, a multifactor working speed model and a joint model for multidimensional ability and multifactor speed were proposed.

First, a motivating example with the EFA of PISA 2012 computer-based mathematics RTs was presented. The results indicate that working speed has a multifactor structure, which is also consistent with the multidimensional structure of ability. Then, two simulation studies were used to evaluate the psychometric properties of the proposed joint model. The results indicate that (1) parameters of the proposed joint model could be well recovered using the proposed Bayesian MCMC approach, (2) misspecifying a multifactor structure of speed has limited effect on the recovery of model parameters, and (3) ignoring the multifactor structure of speed could lead to biased and imprecise estimation, especially for time-related parameters. The PISA 2012 computer-based mathematics RA and RT data were analyzed as well to illustrate the implications and applications of the proposed models. The results show that it is appropriate to consider the multidimensionality of latent ability and the multifactor structure of working speed, simultaneously, in multidimensional tests. Overall, considering the results of EFA, the simulation studies, and the empirical example, there are reasons to believe that the kernel hypothesis of this study is valid and the proposed model can reasonably jointly analyze RA and RTs in multidimensional tests.

The work presented in this article is only a first attempt to deal with the variable speed across dimensions of ability. Despite promising results, further exploration is encouraged. First, the proposed MLRT model is an extension of the classical lognormal RT model (van der Linden, 2006). Thus, there are some limitations of the current model. For instance, it assumes that RA and RTs are conditionally independent given all person parameters (Meng et al., 2015; Bolsinova and Maris, 2016); that after log-transformation, the log RTs follow a normal distribution (Klein Entink et al., 2009b); and that all respondents apply the same problem-solving strategy throughout the whole test (Wang and Xu, 2015).

Second, although the proposed model takes into account the differences in working speed across different dimensions of ability, it still assumes that the working speed of a respondent is constant on items within the same dimension. In future studies, this hypothesis can be further relaxed; that is, each respondent could be allowed to change his or her working speed in different dimensions, and could also be allowed to adjust his or her working speed within the same dimension according to the order of items.

Third, in the proposed joint model, a multivariate normal distribution was used to describe the relationships among multidimensional ability and multifactor speed. So, the number of total dimensions is twice as many as the number of dimensions that are measured by the test, which may increase the complexity of the model and the computational burden. If the ability and speed can each have a second-order (or bi-factor) structure, not only can the parameter estimation challenge be largely reduced, but the structures of ability and speed can be posited and tested.

Fourth, in this study, only the MR model and the MLRT model were used as measurement models for illustration. Given the “plug-and-play” nature of the hierarchical modeling, various MIRT models and multifactor working speed models can be adopted in the future.

Fifth, applications of the proposed model, such as detecting aberrant responses (e.g., rapid-guessing and cheating) in multidimensional tests, need further investigation.

Moreover, in Bayesian estimation, the prior distribution reflects the data analyst’s beliefs and the known information about the data. In practice, we recommend that the data analyst select appropriate prior distributions based on the actual test scenario rather than copy those given in this study.

Last but not least, only the between-item multidimensional test was considered in this study. For the between-item multidimensional test, it is clear that working speed can vary across items when the items are related to different dimensions. However, the within-item multidimensional test is still possible in reality. For example, when respondents, especially non-native English speakers, take part in the GRE^®^ Subject Test (e.g., Mathematics), at least two abilities are needed: one for understanding the questions (e.g., English reading ability), and one for solving the questions (e.g., the subject ability). Meanwhile, the corresponding two latent speed factors work; one reflects the working speed of reading, and the other one reflects the working speed of problem-solving. The introduction of within-item multidimensionality is bound to increase the complexity of the model and the difficulty of constructing the Q-matrix. Thus, the rationality and necessity of the within-item multifactor working speed model is still an open-ended question needed to be studied in the future.

## Data Availability Statement

Publicly available datasets were analyzed in this study. This data can be found here: http://www.oecd.org/pisa/data/pisa2012database-downloadabledata.htm and http://www.oecd.org/pisa/data/pisa2012database-downloadabledata.htm.

## Author Contributions

PZ contributed to the conception, design, and analysis of data as well as paper drafting and revising the manuscript. HJ contributed to the design and critically revising the manuscript. W-CW contributed to conception, design, and revising the manuscript. KM contributed to the critically revising the manuscript. W-CW contributed to conception, design, and revising the manuscript. KH contributed to the interpretation of data and critically revising the manuscript. All authors contributed to the article and approved the submitted version.

## Conflict of Interest

The authors declare that the research was conducted in the absence of any commercial or financial relationships that could be construed as a potential conflict of interest.

## References

[B1] Adams R. J. Wilson M. Wang W. (1997). The multidimensional random coefficients multinomial logit model. *Appl. Psychol. Meas.* 21 1–23. 10.1177/0146621697211001

[B2] Akaike H. (1974). A new look at the statistical model identification. *IEEE Trans. Automat. Contr.* 19 716–723. 10.1109/TAC.1974.1100705

[B3] Bolsinova M. Maris G. (2016). A test for conditional independence between response time and accuracy. *Br. J. Math. Stat. Psychol.* 69 62–79. 10.1111/bmsp.12059 26059168

[B4] Bolsinova M. Tijmstra J. (2018). Improving precision of ability estimation: getting more from response times. *Br. J. Math. Stat. Psychol.* 71 13–38. 10.1111/bmsp.12104 28635139

[B5] Brooks S. P. Gelman A. (1998). General methods for monitoring convergence of iterative simulations. *J. Comp. Graphical Stat.* 7 434–455. 10.2307/1390675

[B6] De Boeck P. Jeon M. (2019). An overview of models for response times and processes in cognitive tests. *Front. Psychol.* 10:102. 10.3389/fpsyg.2019.00102 30787891 PMC6372526

[B7] Fox J.-P. Entink R. K. Timmers C. (2014). The joint multivariate modeling of multiple mixed response sources: relating student performances with feedback behavior. *Multiv. Behav. Res.* 49 54–66. 10.1080/00273171.2013.843441 26745673

[B8] Fox J.-P. Marianti S. (2016). Joint modeling of ability and differential speed using responses and response times. *Multiv. Behav. Res.* 51 540–553. 10.1080/00273171.2016.1171128 27269482

[B9] Gelfand A. E. Smith A. F. M. (1990). Sampling-based approaches to calculating marginal densities. *J. Am. Stat. Assoc.* 85 398–409. 10.1080/01621459.1990.10476213

[B10] Gelman A. Carlin J. B. Stern H. S. Dunson D. B. Vehtari A. Rubin D. B. (2014). *Bayesian Data Analysis.* New York, NY: Chapman & Hall.

[B11] Hu L. T. Bentler P. M. (1999). Cutoff criteria for fit indexes in covariance structure analysis: conventional criteria versus new alternatives. *Struct. Equ. Modeling* 6 1–55. 10.1080/10705519909540118

[B12] Klein Entink R. H. Fox J.-P. van der Linden W. J. (2009a). A multivariate multilevel approach to the modeling of accuracy and speed of test takers. *Psychometrika* 74 21–48. 10.1007/S11336-008-9075-Y 20037635 PMC2792348

[B13] Klein Entink R. H. van der Linden W. J. Fox J.-P. (2009b). A Box-Cox normal model for response times. *Br. J. Math. Stat. Psychol.* 62 621–640. 10.1348/000711008X374126 19187574

[B14] Man K. Harring J. R. Jiao H. Zhan P. (2019). Joint modeling of compensatory multidimensional item responses and response times. *Appl. Psychol. Meas.* 43 639–654. 10.1177/0146621618824853 31551641 PMC6745633

[B15] Meng X.-B. Tao J. Chang H.-H. (2015). A conditional joint modeling approach for locally dependent item responses and response times. *J. Educ. Meas.* 52 1–27. 10.1111/jedm.12060

[B16] Molenaar D. Oberski D. Vermunt J. De Boeck P. (2016). Hidden markov item response theory models for responses and response times. *Multiv. Behav. Res.* 51 606–626. 10.1080/00273171.2016.1192983 27712114

[B17] Molenaar D. Tuerlinckx F. van der Maas H. L. J. (2015). A generalized linear factor model approach to the hierarchical framework for responses and response times. *Br. J. Math. Stat. Psychol.* 68 197–219. 10.1111/bmsp.12042 25109494

[B18] Muthén L. K. Muthén B. (2019). *Mplus. The Comprehensive Modeling Program for Applied Researchers: User’s Guide.* Los Angeles, CA: Mutheìn & Mutheìn, 5.

[B19] OECD (2013). *PISA 2012 Assessment and Analytical Framework: Mathematics, Reading, Science, Problem Solving and Financial Literacy.* Paris: OECD Publishing. 10.1787/9789264190511-en

[B20] Plummer M. (2015). *JAGS Version 4.0.0 User Manual.* Available online at: http://sourceforge.net/projects/mcmc-jags/ (accessed March 1, 2021).

[B21] Reckase M. (2009). *Multidimensional Item Response Theory.* New York, NY: Springer.

[B22] Rupp A. Templin J. Henson R. (2010). *Diagnostic Measurement: Theory, Methods, and Applications.* New York, NY: Guilford Press.

[B23] Schwarz G. (1978). Estimating the dimension of a model. *Ann. Stat.* 6, 461–464. 10.1214/aos/1176344136

[B24] Steiger J. H. (1990). Structural model evaluation and modification: an interval estimation approach. *Multiv. Behav. Res.* 25 173–180. 10.1207/s15327906mbr2502_4 26794479

[B25] Tatsuoka K. K. (1983). Rule space: an approach for dealing with misconceptions based on item response theory. *J. Educ. Meas.* 20 345–354. 10.1111/j.1745-3984.1983.tb00212.x

[B26] van der Linden W. J. (2006). A lognormal model for response times on test items. *J. Educ. Behav. Stat.* 31 181–204. 10.3102/10769986031002181

[B27] van der Linden W. J. (2007). A hierarchical framework for modeling speed and accuracy on test items. *Psychometrika* 72 287–308. 10.1007/s11336-006-1478-z

[B28] van der Linden W. J. Klein Entink R. Fox J.-P. (2010). IRT parameter estimation with response times as collateral information. *Appl. Psychol. Meas.* 34 327–347. 10.1177/0146621609349800

[B29] Wang C. Chang H. Douglas J. (2013). The linear transformation model with frailties for the analysis of item response times. *Br. J. Math. Stat. Psychol.* 66 144–168. 10.1111/j.2044-8317.2012.02045.x 22506914

[B30] Wang C. Weiss D. J. Su S. (2019). Modeling response time and responses in multidimensional health measurement. *Front. Psychol.* 10:51. 10.3389/fpsyg.2019.00051 30761036 PMC6361798

[B31] Wang C. Xu G. (2015). A mixture hierarchical model for response times and response accuracy. *Br. J. Math. Stat. Psychol.* 68 456–477. 10.1111/bmsp.12054 25873487

[B32] Yan D. Mislevy R. J. Almond R. G. (2003). *Design and Analysis in a Cognitive Assessment* (ETS Research Report Series, RR-03-32). Princeton, NJ: ETS.

[B33] Zhan P. Jiao H. Liao D. (2018). Cognitive diagnosis modeling incorporating item response times. *Br. J. Math. Stat. Psychol.* 71 262–286. 10.1111/bmsp.12114 28872185

