# Supplementary material for: Variable Speed Across Dimensions of Ability in the Joint Model for Responses and Response Times
Source: Front Psychol. 2021 Mar 29;12:469196. doi: 10.3389/fpsyg.2021.469196 (PMC8039373; doi:10.3389/fpsyg.2021.469196)
Supplement: Supplementary file 1 [file Table_1.DOCX]

**Online Appendix**

**Variable Speed Across Dimensions of Ability in the Joint Model for Responses and Response Times**

**Sample JAGS code for the MMJ model**

###########

#N: Sample size

#I: test length

#Q: Q matrix

#Score: Response accuracy

#logT: log response time

#theta.1: the first dimension of latent ability

#tau.1: the first factor of latent speed

#xi: item time-intensity

#omega: item time-kurtosis

#d: item intercept

#person_cov: person variance and covariance matrix

#item_cov: item variance and covariance matrix

###########

MMJ.model

{

for (n in 1:N) {

for (i in 1:I) {

logit(prob[n, i]) <- Q[i,1]*theta.1[n]+Q[i,2]*theta.2[n]+Q[i,3]*theta.3[n]+d[i]

Score[n, i] ~ dbern(prob[n, i])

logT[n, i] ~ dnorm(mu_logt[n,i], epsilon[i])

mu_logt[n,i]<-xi[i]-(tau.1[n]*Q[i,1]+tau.2[n]*Q[i,2]+tau.3[n]*Q[i,3])

}}

for (n in 1:N) {

person_parameter[n, 1:6] ~ dmnorm(person_mu[1:6], person_den[1:6, 1:6])

theta.1[n] <- person_parameter[n, 1]

theta.2[n] <- person_parameter[n ,2]

theta.3[n] <- person_parameter[n, 3]

tau.1[n] <- person_parameter[n, 4]

tau.2[n] <- person_parameter[n, 5]

tau.3[n] <- person_parameter[n, 6]

}

for (i in 1:I) {

item_parameter[i, 1:2] ~ dmnorm(item_mu[1:2],item_den[1:2, 1:2])

d[i] <- item_parameter[i, 1]

xi[i] <- item_parameter[i, 2]

den_omega[i] ~ dgamma(1, 1)

omiga[i]<-sqrt(epsilon[i])

Sigma_epsilon[i] <- 1/epsilon[i]

}

person_mu[1] <- 0

person_mu[2] <- 0

person_mu[3] <- 0

person_mu[4] <- 0

person_mu[5] <- 0

person_mu[6] <- 0

R_person[1,1]<-1

R_person[2,2]<-1

R_person[3,3]<-1

R_person[4,4]<-1

R_person[5,5]<-1

R_person[6,6]<-1

R_person[1,2]<-0

R_person[1,3]<-0

R_person[1,4]<-0

R_person[1,5]<-0

R_person[1,6]<-0

R_person[2,3]<-0

R_person[2,4]<-0

R_person[2,5]<-0

R_person[2,6]<-0

R_person[3,4]<-0

R_person[3,5]<-0

R_person[3,6]<-0

R_person[4,5]<-0

R_person[4,6]<-0

R_person[5,6]<-0

R_person[2,1]<-0

R_person[3,1]<-0

R_person[4,1]<-0

R_person[5,1]<-0

R_person[6,1]<-0

R_person[3,2]<-0

R_person[4,2]<-0

R_person[5,2]<-0

R_person[6,2]<-0

R_person[4,3]<-0

R_person[5,3]<-0

R_person[6,3]<-0

R_person[5,4]<-0

R_person[6,4]<-0

R_person[6,5]<-0

person_den[1:6, 1:6] ~ dwish(R_person[1:6, 1:6], 6)

person_cov[1:6, 1:6] <- inverse(person_den[1:6, 1:6])

item_mu[1] ~ dnorm(0, 0.5)

item_mu[2] ~ dnorm(4.3, 0.5)

R_item[1, 1] <- 1

R_item[1, 2] <- 0

R_item[2, 1] <- 0

R_item[2, 2] <- 1

item_den[1:2, 1:2] ~ dwish(R_item[1:2, 1:2], 2)

item_cov[1:2, 1:2] <- inverse(item_den[1:2, 1:2])

}

**Section S1: Simulation Study 2**

**Data Generation and Analysis**

In simulation study 2, two factors were manipulated including (a) the data generation model: the MMJ model and the MSJ model and (b) the number of dimensions of ability: *K* = 3 and 5. In each condition, the MMJ model and the MSJ model were used to fit the data. The test length and sample size were fixed at 30 and 1,000, respectively. The correlation coefficient between latent ability and its corresponding latent speed was fixed at –0.4. The generation methods of item parameters and person parameters were consistent with those in Simulation Study 1. Thirty data sets were generated in each condition. Analysis processes were identical to those in Simulation Study 1. Additionally, the DIC and WAIC were computed for model selection. A smaller value of these two indicates a better model-data fit.

**Results**

Table S1 presents the recovery of item parameters. First, when the MSJ model was used as the true model, the recovery of the MMJ model was almost identical to that of the MSJ model. By contrast, when the MMJ model was used as the true model, only the recovery of item time-discrimination was affected. Specifically, using the MSJ model to analyze data generated by the MMJ model lead to more bias (underestimate, strictly) and higher RMSE of the item time-discrimination.

Tables S2 and S3 present the recovery of latent ability and latent speed, respectively. First, in terms of latent ability, as the same measurement model for RA was used, the recovery of the MMJ model is almost identical to that of the MSJ model, regardless of the true model. Secondly, the main difference between these two models reflected in the recovery of latent speed. Specifically, when the MSJ model was used as the true model, slightly higher RMSE and slightly smaller Cor were obtained by using the MMJ model than using the true model as the analysis model. By contrast, however, when the MMJ model was used as the true model, the recovery of MSJ model is significantly worse than that of the true model, in terms of the RMSE and Cor.

Table S4 presents the recovery of the item mean vector and item variance-covariance. The difference between the two models was small, across all conditions. Tables S5 and S6 present the recovery of variances of person parameters. The difference between the recovery of two models was greater in latent speed than in latent ability. The recovery of the true model itself is better, across all conditions. Although misspecifying a multifactor structure of latent speed produces multiple estimated latent speeds, the correlation coefficients among them were quite high (i.e., around .958 for three dimensions and around .944 for five dimensions). Such a high correlation coefficient can alert data analysts that there may be only one dimension in the latent structure.

Overall, the results of simulation study 2 indicated that (1) misspecifying a multifactor structure of latent speed has limited effect on the recovery of model parameters; and (2) ignoring the multifactor structure of latent speed would lead to biased and imprecise estimation, especially for time-related parameters.

**Table S1**

*Recovery of Item Parameters in Simulation Study 2.*

| True Model | *K* | Analysis Model | Mean Bias | | | Mean RMSE | | | Cor | | |
| --- | --- | --- | --- | --- | --- | --- | --- | --- | --- | --- | --- |
|  |  |  | *d* | ξ | ω | *d* | ξ | ω | *d* | ξ | ω |
| MSJ | 3 | MSJ | –0.001 | 0.000 | –0.009 | 0.074 | 0.016 | 0.046 | 0.997 | 0.999 | NA |
|  |  | MMJ | –0.001 | 0.000 | 0.006 | 0.074 | 0.016 | 0.047 | 0.997 | 1.000 | NA |
|  | 5 | MSJ | 0.001 | 0.000 | –0.007 | 0.073 | 0.016 | 0.044 | 0.997 | 1.000 | NA |
|  |  | MMJ | 0.001 | 0.000 | 0.010 | 0.074 | 0.016 | 0.050 | 0.997 | 1.000 | NA |
| MMJ | 3 | MSJ | 0.001 | –0.001 | –0.235 | 0.075 | 0.016 | 0.238 | 0.997 | 1.000 | NA |
|  |  | MMJ | 0.001 | –0.001 | –0.007 | 0.075 | 0.016 | 0.047 | 0.997 | 1.000 | NA |
|  | 5 | MSJ | –0.003 | 0.001 | –0.270 | 0.075 | 0.015 | 0.273 | 0.997 | 1.000 | NA |
|  |  | MMJ | –0.003 | 0.000 | –0.007 | 0.076 | 0.015 | 0.051 | 0.997 | 1.000 | NA |

*Note*, MSJ = multidimensional-single-factor joint model; MMJ = multidimensional-multifactor joint model; K = number of dimensions of ability; *d* = item intercept/easiness; ξ = item time-intensity; ω = item time-discrimination; Mean Bias = mean bias across all items; Mean RMSE = mean root mean square error across all items; Cor = correlation between estimated and true values; Cor of ω is NA because of the variance of true ω is zero.

**Table S2**

*Recovery of Multidimensional Latent Ability in Simulation Study 2.*

| True Model | *K* | Analysis Model | Mean Bias | | | | | Mean RMSE | | | | | Cor | | | | |
| --- | --- | --- | --- | --- | --- | --- | --- | --- | --- | --- | --- | --- | --- | --- | --- | --- | --- |
|  |  |  | θ_1_ | θ_2_ | θ_3_ | θ_4_ | θ_5_ | θ_1_ | θ_2_ | θ_3_ | θ_4_ | θ_5_ | θ_1_ | θ_2_ | θ_3_ | θ_4_ | θ_5_ |
| MSJ | 3 | MSJ | 0.000 | 0.000 | 0.000 |  |  | 0.494 | 0.492 | 0.492 |  |  | 0.867 | 0.868 | 0.869 |  |  |
|  |  | MMJ | 0.000 | 0.000 | 0.000 |  |  | 0.494 | 0.492 | 0.492 |  |  | 0.867 | 0.868 | 0.869 |  |  |
|  | 5 | MSJ | 0.001 | 0.000 | 0.000 | 0.000 | 0.000 | 0.529 | 0.530 | 0.531 | 0.528 | 0.526 | 0.847 | 0.846 | 0.845 | 0.847 | 0.849 |
|  |  | MMJ | 0.000 | 0.000 | 0.000 | 0.000 | 0.000 | 0.530 | 0.532 | 0.532 | 0.528 | 0.527 | 0.846 | 0.845 | 0.845 | 0.847 | 0.848 |
| MMJ | 3 | MSJ | 0.000 | 0.000 | 0.000 |  |  | 0.490 | 0.488 | 0.489 |  |  | 0.870 | 0.871 | 0.870 |  |  |
|  |  | MMJ | 0.000 | 0.000 | 0.000 |  |  | 0.491 | 0.489 | 0.490 |  |  | 0.869 | 0.870 | 0.869 |  |  |
|  | 5 | MSJ | 0.000 | 0.000 | 0.000 | 0.000 | 0.000 | 0.524 | 0.524 | 0.522 | 0.524 | 0.525 | 0.849 | 0.849 | 0.851 | 0.849 | 0.849 |
|  |  | MMJ | 0.000 | 0.000 | 0.000 | 0.000 | 0.000 | 0.528 | 0.528 | 0.526 | 0.529 | 0.529 | 0.846 | 0.847 | 0.848 | 0.847 | 0.846 |

*Note*, MSJ = multidimensional-single-factor joint model; MMJ = multidimensional-multifactor joint model; K = number of dimensions of ability; θ = latent ability; Mean Bias = mean bias across all persons; Mean RMSE = mean root mean square error across all persons; Cor = correlation between estimated and true values.

**Table S3**

*Recovery of Multifactor Working Speed in Simulation Study 2.*

| True Model | *K* | Analysis Model | Mean Bias | | | | | Mean RMSE | | | | | Cor | | | | |
| --- | --- | --- | --- | --- | --- | --- | --- | --- | --- | --- | --- | --- | --- | --- | --- | --- | --- |
|  |  |  | τ_1_ | τ_2_ | τ_3_ | τ_4_ | τ_5_ | τ_1_ | τ_2_ | τ_3_ | τ_4_ | τ_5_ | τ_1_ | τ_2_ | τ_3_ | τ_4_ | τ_5_ |
| MSJ | 3 | MSJ | 0.000 |  |  |  |  | 0.089 |  |  |  |  | 0.984 |  |  |  |  |
|  |  | MMJ | *0.000* | *0.000* | *0.000* |  |  | *0.097* | *0.097* | *0.097* |  |  | *0.981* | *0.981* | *0.981* |  |  |
|  | 5 | MSJ | 0.000 |  |  |  |  | 0.089 |  |  |  |  | 0.984 |  |  |  |  |
|  |  | MMJ | *0.000* | *0.000* | *0.000* | *0.000* | *0.000* | *0.101* | *0.101* | *0.101* | *0.101* | *0.101* | *0.979* | *0.979* | *0.979* | *0.979* | *0.979* |
| MMJ | 3 | MSJ | 0.000 | 0.000 | 0.000 |  |  | 0.271 | 0.271 | 0.270 |  |  | 0.838 | 0.837 | 0.839 |  |  |
|  |  | MMJ | 0.000 | 0.000 | 0.000 |  |  | 0.144 | 0.144 | 0.146 |  |  | 0.957 | 0.957 | 0.956 |  |  |
|  | 5 | MSJ | 0.000 | 0.000 | 0.000 | 0.000 | 0.000 | 0.294 | 0.293 | 0.294 | 0.294 | 0.294 | 0.805 | 0.806 | 0.805 | 0.805 | 0.805 |
|  |  | MMJ | 0.000 | 0.000 | 0.000 | 0.000 | 0.000 | 0.175 | 0.175 | 0.176 | 0.177 | 0.176 | 0.936 | 0.935 | 0.935 | 0.934 | 0.935 |

*Note*, MSJ = multidimensional-single-factor joint model; MMJ = multidimensional-multifactor joint model; K = number of dimensions of ability; τ = working speed factor; Mean Bias = mean bias across all persons; Mean RMSE = mean root mean square error across all persons; Cor = correlation between estimated and true values; Italic represents multiple estimates to a true value; Underline represents one estimate to multiple true values.

**Table S4**

*Recovery of Item Mean Vector and Item Variance-Covariance in Simulation Study 2.*

| True Model | *K* | Analysis Model | Bias | | | | | RMSE | | | | |
| --- | --- | --- | --- | --- | --- | --- | --- | --- | --- | --- | --- | --- |
|  |  |  | σd2 | σ*_d_*_ξ_ | σ_ξ_2 | μd | μ_ξ_ | σd2 | σ*_d_*_ξ_ | σ_ξ_2 | μd | μ_ξ_ |
| MSJ | 3 | MSJ | 0.068 | 0.008 | 0.044 | 0.008 | 0.003 | 0.077 | 0.010 | 0.044 | 0.012 | 0.004 |
|  |  | MMJ | 0.066 | 0.007 | 0.044 | 0.008 | 0.003 | 0.076 | 0.009 | 0.044 | 0.010 | 0.004 |
|  | 5 | MSJ | 0.057 | 0.010 | 0.045 | 0.014 | 0.003 | 0.062 | 0.012 | 0.045 | 0.018 | 0.004 |
|  |  | MMJ | 0.050 | 0.009 | 0.045 | 0.015 | 0.003 | 0.057 | 0.011 | 0.045 | 0.019 | 0.004 |
| MMJ | 3 | MSJ | 0.063 | 0.007 | 0.044 | 0.013 | 0.003 | 0.071 | 0.009 | 0.045 | 0.016 | 0.003 |
|  |  | MMJ | 0.062 | 0.006 | 0.044 | 0.013 | 0.003 | 0.069 | 0.008 | 0.044 | 0.016 | 0.004 |
|  | 5 | MSJ | 0.069 | 0.011 | 0.046 | 0.013 | 0.003 | 0.076 | 0.012 | 0.046 | 0.017 | 0.003 |
|  |  | MMJ | 0.053 | 0.008 | 0.045 | 0.013 | 0.002 | 0.060 | 0.010 | 0.045 | 0.017 | 0.003 |

*Note*, MSJ = multidimensional-single-factor joint model; MMJ = multidimensional-multifactor joint model; K = number of dimensions of ability; σ*_d_*^2^ = variance of item intercept/easiness; σ_ξ_^2^ = variance of item time-intensity; σ*_d_*_ξ_ = covariance of item intercept and item time-intensity; RMSE = mean root mean square error.

**Table S5**

*Recovery of the Variance of Latent Ability in Simulation Study 2.*

| True Model | K | Analysis Model | Bias | | | | | RMSE | | | | |
| --- | --- | --- | --- | --- | --- | --- | --- | --- | --- | --- | --- | --- |
|  |  |  | σ_θ1_^2^ | σ_θ2_^2^ | σ_θ3_^2^ | σ_θ4_^2^ | σ_θ5_^2^ | σ_θ1_^2^ | σ_θ2_^2^ | σ_θ3_^2^ | σ_θ4_^2^ | σ_θ5_^2^ |
| MSJ | 3 | MSJ | 0.011 | –0.008 | –0.003 |  |  | 0.050 | 0.068 | 0.073 |  |  |
|  |  | MMJ | 0.002 | –0.015 | –0.011 |  |  | 0.048 | 0.068 | 0.074 |  |  |
|  | 5 | MSJ | –0.017 | –0.031 | –0.011 | –0.003 | –0.014 | 0.111 | 0.088 | 0.087 | 0.084 | 0.085 |
|  |  | MMJ | –0.043 | –0.058 | –0.038 | –0.031 | –0.040 | 0.119 | 0.102 | 0.090 | 0.091 | 0.092 |
| MMJ | 3 | MSJ | 0.001 | 0.014 | –0.028 |  |  | 0.059 | 0.059 | 0.055 |  |  |
|  |  | MMJ | –0.009 | 0.003 | –0.040 |  |  | 0.060 | 0.057 | 0.063 |  |  |
|  | 5 | MSJ | –0.026 | 0.009 | –0.028 | –0.043 | 0.000 | 0.075 | 0.097 | 0.077 | 0.081 | 0.076 |
|  |  | MMJ | –0.040 | –0.023 | –0.030 | –0.044 | –0.022 | 0.079 | 0.097 | 0.091 | 0.097 | 0.074 |

*Note*, MSJ = multidimensional-single-factor joint model; MMJ = multidimensional-multifactor joint model; K = number of dimensions of ability; σ_θ_^2^ = variance of latent ability; RMSE = mean root mean square error.

**Table S6**

*Recovery of the Variance of Latent Speed in Simulation Study 2.*

| True Model | K | Analysis Model | Bias | | | | | RMSE | | | | |
| --- | --- | --- | --- | --- | --- | --- | --- | --- | --- | --- | --- | --- |
|  |  |  | σ_τ1_^2^ | σ_τ2_^2^ | σ_τ3_^2^ | σ_τ4_^2^ | σ_τ5_^2^ | σ_τ1_^2^ | σ_τ2_^2^ | σ_τ3_^2^ | σ_τ4_^2^ | σ_τ5_^2^ |
| MSJ | 3 | MSJ | 0.001 |  |  |  |  | 0.004 |  |  |  |  |
|  |  | MMJ | *0.007* | *0.008* | *0.008* |  |  | *0.009* | *0.009* | *0.010* |  |  |
|  | 5 | MSJ | 0.002 |  |  |  |  | 0.003 |  |  |  |  |
|  |  | MMJ | *0.013* | *0.011* | *0.011* | *0.013* | *0.015* | *0.015* | *0.013* | *0.013* | *0.015* | *0.016* |
| MMJ | 3 | MSJ | –0.068 | –0.068 | –0.068 |  |  | 0.068 | 0.068 | 0.068 |  |  |
|  |  | MMJ | 0.003 | 0.002 | 0.000 |  |  | 0.006 | 0.005 | 0.005 |  |  |
|  | 5 | MSJ | –0.081 | –0.081 | –0.081 | –0.081 | –0.081 | 0.081 | 0.068 | 0.068 | 0.068 | 0.068 |
|  |  | MMJ | 0.000 | 0.002 | 0.003 | 0.001 | 0.000 | 0.006 | 0.008 | 0.009 | 0.008 | 0.006 |

*Note*, MSJ = multidimensional-single-factor joint model; MMJ = multidimensional-multifactor joint model; K = number of dimensions of ability; σ_τ_^2^ = variance of latent speed; RMSE = mean root mean square error; Italic represents multiple estimates to a true value; Underline represents one estimate to multiple true values.

**Section S2: Results of Empirical Study**

To explore the relationships among multiple latent abilities and latent speeds, Table S7 presents the estimated person variance-covariance matrix. Both high positive correlations were found among the multiple abilities (.80 ~ .85) and the multiple speeds (.75 ~ .89), respectively. And low to moderate negative correlations (–.68 ~ –.29) were found between the abilities and speeds. According to the results of simulation study 2, when the structure of latent speed was misspecified (i.e., the true structure is unidimensional), the correlation coefficients among multiple estimated latent speeds are around .95. In the empirical example, the correlations of such three dimensions of latent speed are around .8, indicating those dimensions are high correlated but still can be separable. Note that the variance of the first latent ability was quite large, indicating all respondents differ greatly in the first dimension.

Table S8 presents the estimates of the item parameters. The estimated mean item easiness and mean time-intensity were –1.360 (SE = .551) and 4.289 (SE = .170), respectively. To further explore the relationship between the item easiness and time-intensity parameters, we present the estimated item variance-covariance matrix in Table S9. The correlation was negative, which implied that test-takers tend to spend longer time on the harder items (i.e., items with lower easiness). This result was consistent with that in the literature (Fox & Marianti, 2016; Meng et al., 2015; van der Linden, 2006; 2007).

**Table S7**

*Estimated Variance-Covariance Matrix for* *Multidimensional Ability and Multifactor Speed.*

|  | θ_1_ | θ_2_ | θ_3_ | τ_1_ | τ_2_ | τ_3_ |
| --- | --- | --- | --- | --- | --- | --- |
| θ_1_ | 11.970 (1.574)  [8.887, 14.230] | 0.800 | 0.821 | –0.683 | –0.290 | –0.385 |
| θ_2_ | 3.936 (0.356)  [3.277, 4.684] | 2.023 (0.175)  [1.695, 2.386] | 0.852 | –0.466 | –0.289 | –0.412 |
| θ_3_ | 2.831 (0.257)  [2.365, 3.379] | 1.209 (0.095)  [1.027, 1.400] | 0.994 (0.113)  [0.789, 1.241] | –0.618 | –0.394 | –0.580 |
| τ_1_ | –1.316 (0.128)  [–1.577, –1.078] | –0.369 (0.036)  [–0.440, –0.300] | –0.343 (0.032)  [–0.408, –0.283] | 0.310 (0.016)  [0.279, 0.344] | 0.893 | 0.749 |
| τ_2_ | –0.450 (0.078)  [–0.607, –0.299] | –0.185 (0.028)  [–0.239, –0.131] | –0.177 (0.023)  [–0.221, –0.133] | 0.184 (0.010)  [0.166, 0.203] | 0.202 (0.009)  [0.185, 0.221] | 0.849 |
| τ_3_ | –0.722 (0.099)  [–0.920, –0.532] | –0.317 (0.034)  [–0.385, –0.252] | –0.313 (0.031)  [–0.374, –0.256] | 0.226 (0.012)  [0.204, 0.250] | 0.207 (0.009)  [0.189, 0.226] | 0.294 (0.013)  [0.268, 0.321] |

*Note*, Standard error in parentheses; 95% Bayesian credibility interval in square brackets.

**Table S8**

*Estimated Item Parameters for the Released 2012 PISA Computer-Based Mathematics Items.*

| Item | *d* | ξ | ω |
| --- | --- | --- | --- |
| 1 | –4.507 (0.266)  [–5.048, –3.982] | 4.473 (0.019)  [4.434, 4.511] | 1.847 (0.042)  [1.766, 1.932] |
| 2 | –3.406 (0.222)  [–3.853, –2.972] | 4.632 (0.018)  [4.596, 4.668] | 2.037 (0.052)  [1.938, 2.144] |
| 3 | –2.388 (0.094)  [–2.574, –2.206] | 4.779 (0.015)  [4.750, 4.808] | 2.511 (0.056)  [2.404, 2.620] |
| 4 | –0.133 (0.070)  [–0.268, 0.004] | 3.861 (0.017) [3.827, 3.894] | 1.920 (0.038)  [1.846, 1.996] |
| 5 | –1.446 (0.080)  [–1.604, –1.291] | 4.259 (0.016)  [4.227, 4.290] | 2.197 (0.047)  [2.106, 2.289] |
| 6 | –0.420 (0.069)  [–0.556, –0.284] | 3.740 (0.016)  [3.708, 3.772] | 2.105 (0.044)  [2.020, 2.190] |
| 7 | 0.746 (0.064)  [0.623, 0.872] | 4.190 (0.017)  [4.158, 4.222] | 2.533 (0.062)  [2.414, 2.658] |
| 8 | –1.375 (0.072)  [–1.519, –1.237] | 4.523 (0.018)  [4.488, 4.558] | 2.100 (0.047)  [2.009, 2.192] |
| 9 | –1.431 (0.073)  [–1.575, –1.289] | 4.379 (0.020)  [4.340, 4.418] | 1.699 (0.036)  [1.628, 1.770] |

*Note*, Standard error in parentheses; 95% Bayesian credibility interval in square brackets.

**Table S9**

*Estimated Variance-Covariance Matrix of Item Parameters for the Released 2012 PISA Computer-based Mathematics Items.*

|  | *d* | ξ |
| --- | --- | --- |
| *d* | 3.226 (1.992)  [1.167, 8.331] | –0.449 |
| ξ | –0.425 (0.435)  [–1.449, 0.148] | 0.278 (0.175)  [0.103, 0.715] |

*Note*, covariance in lower triangular and correlation coefficient in upper triangular; Standard error in parentheses; 95% Bayesian credibility interval in square brackets.
